# Supplementary material for: Association between the retinal vascular network and retinal nerve fiber layer in the elderly: The Montrachet study
Source: PLoS One. 2020 Oct 21;15(10):e0241055. doi: 10.1371/journal.pone.0241055 (PMC7577490; doi:10.1371/journal.pone.0241055)
Supplement: S1 Table — (DOCX) [file pone.0241055.s001.docx]

## S1 Table. Baseline Characteristics Between Participants and Non-participants in the Montrachet Study

| **Baseline characteristics** | **Total,**  ***n* = 1153** | **Participants,**  ***n* = 970** | **Non-participants,**  ***n* = 183** | **P-value** |
| --- | --- | --- | --- | --- |
| Age, years |  |  |  |  |
| <80 | 400 (34.69) | 351 (36.18) | 49 (26.78) | <0.001 |
| 80–85 | 486 (42.15) | 416 (42.89) | 69 (37.70) |  |
| >85 | 267 (23.16) | 203 (20.93) | 65 (35.52) |  |
| Sex |  |  |  |  |
| Female | 723 (62.71) | 617 (63.61) | 106 (57.92) | 0.145 |
| Smoking status, self-declared | |  |  |  |
| Smokers, current and past | 420 (42.64) | 384 (42.86) | 36 (40.45) | 0.661 |
| Diabetes, self-declared |  |  |  |  |
| Yes | 121 (12.90) | 111 (12.73) | 10 (11.90) | 0.775 |
| Treatment for systemic hypertension | |  |  |  |
| Yes for < 10 years | 382 (37.67) | 256 (27.71) | 30 (33.33) | 0.487 |
| Yes for ≥ 10 years | 286 (28.21) | 349 (37.77) | 33 (36.67) |  |
| Cholesterol-lowering drug use | |  |  |  |
| Yes | 700 (68.56) | 644 (69.10) | 56 (62.92) | 0.230 |
| MACCE |  |  |  |  |
| Yes | 111 (11.06) | 102 (11.17) | 9 (9.89) | 0.710 |
| HSCORE | 4.07 ± 4.06 | 4.03 ± 4.04 | 4.05 ± 4.03 | 0.271 |
| Axial length, mm | 23.26 ± 1.25 | 23.21 ± 1.21 | 23.76 ± 1.52 | <0.001 |

P-value was calculated between participants and non-participants. The results are displayed as *n* (%) for categorical variables and mean ± standard deviation for continuous variables. MACCE, major adverse cardiovascular or cerebrovascular events; HSCORE, Heart SCORE. Available data for each variable, smoking status *n* = 985; diabetes *n* = 938; treatment for systemic hypertension *n* = 1014; cholesterol-lowering drug use *n* = 1021; MACCE *n* = 1004; HSCORE *n* = 951; axial length *n* = 878.
