# Supplementary material for: Association between the retinal vascular network and retinal nerve fiber layer in the elderly: The Montrachet study
Source: PLoS One. 2020 Oct 21;15(10):e0241055. doi: 10.1371/journal.pone.0241055 (PMC7577490; doi:10.1371/journal.pone.0241055)
Supplement: S2 Table — (DOCX) [file pone.0241055.s002.docx]

## S2 Table. Associations Between Retinal Vascular Parameters and Sectorial Temporal Retinal Nerve Fiber Layer Thickness

|  | **Crude associations** | |  | | **Age, sex-adjusted** | | | |  | **Multivariable-adjusted**^*^ | | |
| --- | --- | --- | --- | --- | --- | --- | --- | --- | --- | --- | --- | --- |
| **Retinal vascular parameters**  (**per SD decrease)** | **β (SE)** | **P-value** |  | | **β (SE)** | | **P-value** | |  | **β (SE)** | **Unadjusted P-value** | **FDR Adjusted P-value** |
| Caliber, μm |  |  |  |  | |  | |  | |  |  |  |
| Six largest arterioles in zone B | 1.74 (0.53) | 0.001 |  | 1.84 (0.53) | | <0.001 | |  | | 2.20 (0.62) | <0.001 | 0.009 |
| Six largest veins in zone B | 1.08 (0.53) | 0.041 |  | 1.08 (0.53) | | 0.040 | |  | | 1.26 (0.61) | 0.041 | 0.048 |
| Six largest arterioles in zone C | 1.56 (0.53) | 0.003 |  | 1.67 (0.54) | | 0.002 | |  | | 1.60 (0.62) | 0.009 | 0.039 |
| Six largest veins in zone C | 0.77 (0.53) | 0.146 |  | 0.75 (0.53) | | 0.158 | |  | | 0.75 (0.62) | 0.226 | 0.319 |
| Fractal dimension |  |  |  |  | |  | |  | |  |  |  |
| Total zone C | 1.21 (0.50) | 0.015 |  | 1.29 (0.50) | | 0.010 | |  | | 1.33 (0.57) | 0.020 | 0.045 |
| Arterioles zone C | 1.41 (0.49) | 0.004 |  | 1.48 (0.49) | | 0.002 | |  | | 1.39 (0.56) | 0.013 | 0.039 |
| Veins zone C | 0.56 (0.51) | 0.266 |  | 0.62 (0.50) | | 0.218 | |  | | 0.66 (0.57) | 0.248 | 0.319 |
| Vascular tortuosity |  |  |  |  | |  | |  | |  |  |  |
| Simple tortuosity, vessels, × 10^4^ | 0.83 (1.12) | 0.456 |  | 0.70 (1.12) | | 0.531 | |  | | 0.90 (1.25) | 0.473 | 0.532 |
| Curvature tortuosity, vessels, × 10^4^ | -0.04 (0.49) | 0.940 |  | -0.11 (0.49) | | 0.824 | |  | | 0.21 (0.54) | 0.698 | 0.698 |

SD, standard deviation; SE, standard error. FDR, false discovery rate. 172 observations were deleted due to missing axial length variable.

^*^ Adjusted for age, sex, axial length, diabetes, and systemic hypertension
