# Supplementary material for: Association between the retinal vascular network and retinal nerve fiber layer in the elderly: The Montrachet study
Source: PLoS One. 2020 Oct 21;15(10):e0241055. doi: 10.1371/journal.pone.0241055 (PMC7577490; doi:10.1371/journal.pone.0241055)
Supplement: S3 Table — (DOCX) [file pone.0241055.s003.docx]

## S3 Table. Associations Between Retinal Vascular Parameters and Sectorial Nasal Retinal Nerve Fiber Layer Thickness

|  | **Crude associations** | |  | **Age, sex-adjusted** | | | |  | | **Multivariable-adjusted*** | | |
| --- | --- | --- | --- | --- | --- | --- | --- | --- | --- | --- | --- | --- |
| **Retinal vascular parameters**  (**per SD decrease)** | **β (SE)** | **P-value** |  | **β (SE)** | | **P-value** | |  | | **β (SE)** | **Unadjusted P-value** | **FDR Adjusted P-value** |
| Caliber, μm |  |  |  |  |  | |  | |  | |  |  |
| Six largest arterioles in zone B | -3.68 (0.61) | <0.001 |  | -3.58 (0.62) | <0.001 | |  | | -1.98 (0.70) | | 0.005 | 0.015 |
| Six largest veins in zone B | -4.23 (0.60) | <0.001 |  | -4.17 (0.60) | <0.001 | |  | | -2.87 (0.69) | | <0.001 | <0.001 |
| Six largest arterioles in zone C | -3.48 (0.61) | <0.001 |  | -3.37 (0.62) | <0.001 | |  | | -1.88 (0.69) | | 0.007 | 0.016 |
| Six largest veins in zone C | -4.32 (0.60) | <0.001 |  | -4.27 (0.60) | <0.001 | |  | | -2.98 (0.69) | | <0.001 | <0.001 |
| Fractal dimension |  |  |  |  |  | |  | |  | |  |  |
| Total zone C | -2.18 (0.58) | <0.001 |  | -2.08 (0.58) | <0.001 | |  | | -1.34 (0.64) | | 0.037 | 0.051 |
| Arterioles zone C | -2.15 (0.56) | <0.001 |  | -2.06 (0.57) | <0.001 | |  | | -1.30 (0.63) | | 0.039 | 0.051 |
| Veins zone C | -2.12 (0.58) | <0.001 |  | -2.04 (0.59) | <0.001 | |  | | -1.46 (0.64) | | 0.022 | 0.040 |
| Vascular tortuosity |  |  |  |  |  | |  | |  | |  |  |
| Simple tortuosity, vessels, × 10^4^ | -1.18 (1.30) | 0.364 |  | -1.06 (1.30) | 0.417 | |  | | -0.40 (1.41) | | 0.776 | 0.825 |
| Curvature tortuosity, vessels, × 10^4^ | -1.14 (0.57) | 0.799 |  | -0.06 (0.57) | 0.912 | |  | | -0.13 (0.61) | | 0.825 | 0.850 |

SD, standard deviation; SE, standard error. FDR, false discovery rate. 172 observations were deleted due to missing axial length variable.

*Adjusted for age, sex, axial length, diabetes, and systemic hypertension.
