# Supplementary material for: Association between the retinal vascular network and retinal nerve fiber layer in the elderly: The Montrachet study
Source: PLoS One. 2020 Oct 21;15(10):e0241055. doi: 10.1371/journal.pone.0241055 (PMC7577490; doi:10.1371/journal.pone.0241055)
Supplement: Attachment — Submitted filename: Rebuttal letter.docx [file pone.0241055.s004.docx]

Manuscript ID PONE-D-20-00420

Association Between the Retinal Vascular Network and Retinal Nerve Fiber Layer in the Elderly: The Montrachet Study

Thank you for giving us the opportunity to revise this submission following peer review. After consultation with the other authors, we have responded to the reviewers’ constructive suggestions by revising the manuscript as attached. We would like to take the opportunity to respond to the individual points below.

Reviewer #1: In this manuscript, Arnould, et al. report that in elderly study participants without optic neuropathy, thinner retinal nerve fiber layer (RNFL) thickness are associated with reduced caliber of retinal arteries and veins. They also report RNFL thickness is associated with several demographic features including sex, self-declared history of diabetes, and axial length.
Because several authors have published similar results, could the authors highlight or compare and contrast what is novel about their findings? Is it the age of the study participants? Is it the method of analyzing the retinal vessels?

*As suggested, we added these sentences in the revised version of our manuscript as follows (line 265-282), first paragraph of the discussion*

“We found that thinner retinal vessels caliber and reduced fractal dimension of veins in zone C were associated with a thinner global RNFL thickness in non-glaucomatous participants. These results are in keeping with other published data. In a cross-sectional investigation of Asian participants, each SD decrease in retinal arteriolar caliber was associated with a 5.81 μm decrease in the mean global measured RNFL thickness (*P* < 0.001), and each SD decrease in retinal venular caliber was associated with an 8.37 μm decrease in mean global RNFL thickness (*P* < 0.001).^43^ In this study, participants’ mean age was 56.8 years. In a study derived from the Singapore Chinese Eye Study, each SD decrease in the retinal arteriolar caliber was associated with a 1.60 μm decrease in global RNFL thickness (*P* = 0.002), and each SD decrease in venular caliber was associated with a 1.97 μm decrease in average RNFL thickness (*P* < 0.001) (mean age was 53.6 ± 6.7 years).^22^ Finally, this association was also confirmed in a middle-aged Caucasian population, where each 10 μm increase in the retinal arteriolar and venular caliber was associated with a 5.58 μm and a 3.79 μm increase in mean global RNFL thickness, respectively (*P* < 0.001) (mean age was 47.3 ± 0.9 years).^21^ We confirmed in our study the association between RNFL thickness and retinal vessels caliber in an elderly population.”

Half of the study participants reported some degree of hypertension. Was there a significant difference in RNFL thickness or retinal vascular metrics for those with hypertension compared to those without?

*We agree with the concern raised by the Reviewer. Indeed, Mauschitz et al. investigated pooled data from 8 European population-based studies (including the Montrachet Study) and showed reduced RNFL in hypertensive patients (Mauschitz et al. Ophthalmology 2018). Moreover, we fully agree with the Reviewer’s comment concerning hypertension and retinal vascular metrics. Ponto et al. showed that systemic hypertension was associated with lower values of arteriolar retinal caliber (Ponto et al. J Hypertension 2017). Our primary objective was to study the association between RNFL and retinal vascular parameters independently of cardiovascular risk factors. We did not perform in our analysis a comparison between participants with and without hypertension regarding their RNFL thickness as we decided to use this variable in our adjusted model. We added the following sentence in the revised version of our manuscript (line 343-347).*

“In contrast to Lee et al., we did not compare RNFL thickness between participants with and without hypertension.^47^ Indeed, hypertension is known to be associated with reduced RNFL and decreased arteriolar retinal caliber.^34,48^ As a consequence, we added hypertension history to the adjusted-multivariable model. The association between hypertension history, RNFL thickness and retinal vascular metrics warrants further studies”

The purpose of the manuscript defined in the introduction is the detailed analysis comparing retinal vasculature to RNFL thickness. I would recommend emphasizing these findings in the conclusion of the abstract which is vaguely written currently.

*We agree with the Reviewer and we modified the conclusion of the abstract as follows (line 46-47).*

“In this study, we found that decreased retinal vessels caliber was associated with a decreased RNFL thickness in the elderly without optic neuropathy.”

Also, the discussion should be re-organized to focus on retinal vasculature metrics association with RNFL thickness and then followed by RNFL associations with demographic features.

*We thank the Reviewer for emphasizing this crucial point. We re-organized the discussion as mentioned (line 265-282) and (line 324-347).*

What was the mean RNFL? How does this compare to other studies?

*The mean RNFL of the study participants was 92.08 ± 13.29 μm. This result was similar to other population-based studies. The mean RNFL thickness in the Beijing Eye Study was 103.2 ± 12.6 µm (66.2 ± 9.9 years) (YA Xing Wang et al. PlosOne 2013). The mean RNFL thickness in the Alienor study was 86.8 ± 13.7 µm (81.6 ± 4.2 years) (*[*Juan Luis Méndez-Gómez*](https://pubmed.ncbi.nlm.nih.gov/?term=M%C3%A9ndez-G%C3%B3mez+JL&cauthor_id=30646353) *et al. JAMA Netw Open 2018). We added the following sentence in the revised version of our manuscript (line 196)*

“The mean RNFL of the study participants was 92.08 ± 13.29 μm”

Could they authors explain their low rate (10 cases out of 1153) of age-related macular degeneration compared to reported prevalence data (Prevalence and incidence of age-related macular degeneration in Europe: a systematic review and meta-analysis. British Journal of Ophthalmology; 11 November 2019)?

*We thank the Reviewer for highlighting this point. Prevalence of age-related macular degeneration (AMD) in the Montrachet study was reported (Creuzot-Garcher et al. Acta Ophthalmologica 2015). The prevalence is lower compared to reported prevalence data because we investigated a white, urban, and generally healthy population with high economic status. Moreover, in this study, we only excluded participants with late AMD and modification of the vascular architecture in zone B and C attested by a trained grader. As suggested by the Reviewer, we added the following statement in the revised version of our manuscript (line 162-164) and we modified the caption of the Figure.*

*“*Patients presenting with late age-related macular degeneration (AMD) and modification of the vascular architecture in zone B and C, attested by a trained grader, were not retained for the analysis.”

Reviewer #2: Arnould et al. present a manuscript assessing the relationship of arterioles and venules to the RNFL thickness measurements. As noted by the authors, many studies have recently been published showing a link between the RNFL and ocular, systemic and neurodegenerative diseases. Their question, of how this layer is related in the elderly and with respect to the vasculature is an important component to this ongoing research.

This manuscript looks specifically at the relationship of different retinal vasculature morphology metrics (on a fundus image) to the thickness of the RNFL at 3.5mm from the optic disc. They find that arteriole and venous vessel caliber is related to RNFL thickness along with age. While this paper is interesting and normative data is needed to understand how aging affects the retina, this author has several concerns that need to be addressed.

Major Issues:
1. The inclusion of subjects with diabetes means that the relationship reported isn’t purely based on age although the authors do adjust for this by using diabetes (and hypertension as well) as a factor in one of their linear regressions. However, nothing is said about the prevalence of retinopathy (which can happen with both diabetes and hypertension). Additionally, there’s also a known relationship between diabetic retinopathy and the length of time someone has diabetes. Yet none of these things are taken into account leading this reviewer to wonder how the regression and these relationships would change. This reviewer would appreciate a deeper examination of subjects with diabetes and a better understanding of that population’s medical information.

*We fully understand this comment. As reported in our previous study (Creuzot-Garcher et al. Acta Ophthalmologica 2015), there were 9 participants of the Montrachet study with an history of diabetic retinopathy. In our study, we found in our database that only 2 diabetic patients presented with diabetic retinopathy. We decided not to exclude these patients because diabetes was chosen to be an adjustment variable in the linear regression. The primary objective of our study was to investigate the association between retinal nerve fiber layer thickness and retinal vascular architectural parameters independently of cardiovascular risk factors. Unfortunately, we did not collect information about the duration of the diabetes and the glycemic status. However, participants in the Montrachet study were healthy volunteers and their cardiovascular risk factors were well monitored.*

*We added the following statement in the revised version of our manuscript.*

*Line 195-196*

“Two participants with diabetes and diabetic retinopathy were kept in the analysis.”

*Line 337-338*

“In the present study, we did not collect information about the duration of the diabetes and glycemic stability. These parameters should be taken into account in future studies.”

Additionally, the exclusion criteria does not mention how other ocular diseases were treated in terms of including or excluding those populations (i.e. sickle cell disease, Macular Telangiectasia etc.).

*Participants ophthalmic history were collected in the same way for the entire population of the Montrachet study. In previous studies focusing on retinal history in the Montrachet study (Ben Ghezala et al. Retina 2020 and Gabrielle et al. IOVS 2019), we did not find other ocular vascular diseases.*

2. Was ocular magnification taken into account when measuring 3.5 mm away from the optic disc? While axial length itself does not impact thickness measurements (PMID: 30025118), it does impact the placement of where these measurements take place if from a set distance. With longer axial lengths, the circle, if ocular magnification is not taken into account, will cover a larger retinal distance than other subjects with short axial lengths. This leads to increased variation in RNFL thickness measurements which may impact the analysis. Please clarify if ocular magnification was corrected for. If not, it would be preferable for the authors to correct for ocular magnification so that 3.5 mm measurements were the same for each individual. If this is not possible, the lack of correction should be noted in the limitations paragraph.

*We agree with the concerns raised by the Reviewer. We added the following statement in the revised version of our manuscript (line 377-380).*

“Fourth, we did not take into account the ocular magnification in the SIVA measurements. It could increase variation in RNFL thickness around the 3.5-mm-diameter measurements which may impact the analysis for extreme axial length eyes.”

3. Table 1: Stated both in the table and throughout the paper, it says that 970 subjects fit the criteria for the study yet there are additional n values in the same column as the baseline characteristic with no mention of what they mean.

*We thank the Reviewer for highlighting this point. The n values in the first column of Table 1 and Table 2 correspond to the available data for each variable. We agree with the Reviewer that additional n values in the same column with no clear explanation is confusing. Therefore, we placed the n values in the notes under Table 1 and Table 2 to avoid any confusion.*

If only 878 subjects of the 970 subjects had axial length measurements, would this not also impact your findings for the regression?

*We fully agree with Reviewer’s comment. Missing data represent less than 10% of our dataset. As a consequence, we did not perform a multiple imputation method. However, it could affect the regression results and it could reduce the statistical power of our associations.*

Given that some of these factors (such as axial length) are known to impact RNFL thickness, why include these subjects?

*Axial length is a known confounder factor between all retinal vascular parameters and also for RNFL thickness. In this study, we kept the axial length as an adjustment variable in order to investigate the relationship between retinal vascular parameters and RNFL thickness irrespectively of the ametropia.*

Please clarify what the n values for the table mean. If subjects were included without a full set of data as presented, including them for the regression may be misleading and the authors should consider removing these subjects from their analysis.

*As suggested by the Reviewer we removed the participants with missing axial length variable in the multivariable models and a note has been written under the Tables in the revised version of our manuscript.*
4. This reviewer appreciates your transparency with the various regressions that you ran. However, with reading this paper, it is often difficult to remember the central point is to show that even with age, the relationship between retinal vasculature and RNFL thickness holds. If the authors could work to improve the clarity of their writing and work to focus the results in particular, this reviewer thinks it would read significantly easier and the main points would come across much stronger rather than the potential of readers to be caught in the details.

*Thank you to the Reviewer for emphasizing this crucial point. We re-organized the discussion as mentioned. (line 265-282) and (line 324-347).*

Minor Issues
1. Line 59: The sentence states “using fundus and other techniques”. Please expand on these other techniques and discuss the benefits of the fundus over these other techniques.

*We thank the Reviewer for highlighting this point. It is possible to investigate the human microcirculation network with noninvasive devices such as fundus photographs, capillaroscopy, renal doppler or brain imaging. In the Montrachet study, participants benefited from an exhaustive eye examination. Fundus photographs were performed for each participant. Compared to the other techniques, fundus photographs are less expensive, and it can be performed by a technician or a nurse. Moreover, semi-automated software based on fundus are available to thoroughly describe the retinal vascular network with quantitative metrics. Finally, it has been shown that retinal vascular network is reliable to assess the systemic vascular status (Arnould et al. PLoS One 2018). We deleted “other techniques” in the revised manuscript (line 65).*

2. Lines 64-69: Multiple different software is cited but nothing is stated about why this goes into the introduction. I would expect a reason for why SIVA was used if this is brought up in the introduction over the others, or is the benefit of a fundus that there are these automated techniques?

*We fully agree with the Reviewer’s comment. We used SIVA software because at the time of the data collection in the Montrachet study (2009-2013), it was the only software that was able to quantify complex vascular parameters such as fractal dimension and tortuosity. Other softwares were only focused on retinal caliber. We deleted information about other softwares in the introduction of the revised manuscript (line 62-70).*

3. Line 107-109: The sentence about the technicians controlling for image quality and segmentation is ambiguous. Was there an image quality metric with a cut off, were the technicians given any information or guidance or were these metrics purely based on their expertise?

*We thank the reviewer for highlighting this point. We modified the following statement in the revised version of our manuscript (line 121-122).*

“Signal strength and the quality of segmentation were controlled by technicians based on their expertise and training”

4. Is it appropriate to keep p < 0.05 for all tests given the number of statistical tests done? This reviewer would suggest consulting with a biostatistician (if not already done) to make sure that the analysis provided is accurate and that the p-values do not need adjustment given the number of analyses this paper employs.

*We agree with the Reviewer* *that, given the number of statistical tests in this study, keeping p < 0.05 for all tests is not appropriate. As suggested by the Reviewer, we used the procedure described by Benjamini and Hochberg (False Discovery Rate correction) (*Benjamin Y et al . J R Stat Soc. 1995*) to correct p values for multiple testing. FDR Adjusted p values are presented in multivariable models for each independent variable in Tables 3,4 and 5 in the revised version of our manuscript. Significant associations remained stable with this correction except the associations between fractal dimension (total zone C and arterioles zone C) and nasal RNFL thickness. We added the following statement in the revised version of our manuscript (line 183-185).*

“Given the number of statistical tests in this study, we used the procedure of False Discovery Rate (FRD) correction to correct p values for multiple testing.^31^”
