# Supplementary material for: Association between the retinal vascular network and retinal nerve fiber layer in the elderly: The Montrachet study
Source: PLoS One. 2020 Oct 21;15(10):e0241055. doi: 10.1371/journal.pone.0241055 (PMC7577490; doi:10.1371/journal.pone.0241055)
Supplement: Attachment — Submitted filename: Rebuttal letter R2.docx [file pone.0241055.s005.docx]

Manuscript ID PONE-D-20-00420R1

Association Between the Retinal Vascular Network and Retinal Nerve Fiber Layer in the Elderly: The Montrachet Study

Thank you for giving us the opportunity to revise this submission following peer review. After consultation with the other authors, we have responded to the reviewers’ constructive suggestions by revising the manuscript as attached. We

would like to take the opportunity to respond to the individual points below.

Reviewer #1: Thank you for considering revisions. These analyses are difficult in elderly patients because of the confounding variables. For example, in this study nearly 70% of participants have a history of hypertension. Presence of hypertension, and diabetes, likely affects affect retinal vascular parameters, but this information is not presented in the manuscript.

Table 2 shows the association between RNFL to demographics, lifestyle and clinical characteristics. It would be useful to show associations between retinal vascular parameters to demographics, lifestyle and clinical characteristics as well. I would expect there to be significant associations with hypertension, diabetes and possibly axial length. Increased transparency with the multiple variables involved will help readers understand the complexity associated with these analyses.

*We thank the Reviewer for highlighting this point. We previously presented associations between retinal vascular parameters and demographics, lifestyle and clinical characteristics in our manuscript : Association between the retinal vascular network with Singapore “I” Vessel Assessment (SIVA) software, cardiovascular history and risk factors in the elderly: The Montrachet study. (Arnould L et al. PlosOne April 2018). As suggested by the Reviewer, we found significant associations between retinal vascular parameters and hypoglycemic treatment, history of systemic vascular events and increased Heart Score risk. For greater clarity, we did not repeat these results.*

Could the authors run a separate analysis evaluating for association of RNFL and retinal vasculature parameters after excluding hypertension, diabetes and extreme axial length differences? It is unclear if the numbers would support this evaluation.

*Thank you to the Reviewer for emphasizing this crucial point. We ran a separate analysis in participants without hypertension and diabetes. This analysis is presented in Table 3. We added these sentences in the revised version of our manuscript as follows*

*Line 220-225*

“In participants without hypertension and diabetes (n = 294), after adjustment for age, sex and axial length, each SD decrease in the caliber of the six largest arterioles and veins in zone B and in the caliber of the six largest arterioles and veins in zone C was associated with a decrease in global RNFL thickness (β = −1.88 μm, *P* = 0.0371; β = −3.28 μm, *P* < 0.001; β = −2.02 μm, *P* < 0.001; and β = −3.27 μm, *P* < 0.001, respectively) (Table 3).”

*Line 271-272*

“Moreover, in a subgroup analysis (participants without hypertension or diabetes), we found similar results.”

*Line 306-310*

“However, in the subgroup analysis (Table 3), the association between a lower fractal dimension of veins in zone C and a reduction in mean RNFL thickness is no more significant. We could make the hypothesis that presence of hypertension or diabetes affect first and foremost retinal fractal dimension.”

For the RNFL analysis, this work is contrasted by others because it is an “elderly cohort” – could the authors clearly state the mean age in the results section?

*The mean age in this study was 82.06 (± 3.64) years old. We added this information in the results section as follows (Line 184)*

“The mean RNFL of the study participants was 92.08 ± 13.29 μm and the mean age was 82.06 ± 3.64 years old.”

Is Table 1 necessary? This is essentially comparing cases that were excluded to participants that were included. Why is this relevant to the comparison of RNFL to retinal vascular parameters? If important to include, perhaps these findings could be summarized in the text of the results.

*We agree with the concern raised by the Reviewer. Table 1 is now part of the supporting information. In the revised version of our manuscript, it is reported as S1 Table. We added these sentences in the revised version of our manuscript as follows (Line 180-181)*

“The characteristics of the study population and a comparison between participants and non-participants are summarized in the supporting information (S1 Table).”

*Moreover, we transferred Table 4 and 5 in the supporting information (S2 and S3 Table). We added a caption section as requested in the revised version of our manuscript.*

I would exclude the sentence in the abstract describing thinner global RNFL thickness associated with males, patients with diabetes and longer eyes because these findings are not directly relevant to the purpose of the manuscript.

*We fully agree with the reviewer’s comment. We deleted this sentence in the abstract of the revised manuscript.*

Why was axial length not included in Table 2? Axial length may confound other associations – such as gender differences.

*We agree with the concerns raised by the Reviewer. We added this information in the revised version of our manuscript (Table 1).*

Reviewer #2: In general, the authors have made considerable steps in improving their manuscript. The paper now reads clearer and the main points are not lost. However, there are still several points of concern about this study for this reviewer.

Major Concerns:
1. As previously mentioned by reviewer 1, several authors have published similar studies. While the discussion now clearly compares the various studies that have been published, it still remains unclear what is novel about this study or its contribution to the literature. Please highlight these points in your discussion.

*We thank the reviewer for highlighting this point. We added this sentence in the discussion of the revised manuscript (Line 334-337).*

“Strengths of our study include its large elderly population-based sample, extensive retinal vascular parameters phenotype (caliber, fractal dimension and vascular tortuosity) and a subgroup analysis in participants without hypertension or diabetes.”

2. The exclusion criteria state that subjects with epiretinal membranes and late-stage AMD were excluded due to known alterations these pathologies have on the vasculature architecture in the retina. Yet the same argument can be made for subjects with hypertension or with diabetes. Based on this, was the exclusions of the subjects based on the fact that those with ERMs or AMD had primarily ocular diseases and therefore the changes in the retinal vasculature had nothing to do with the cardio-vasculature system or was it truly because the vasculature in the retina is altered? If it’s the former, please update the section of the manuscript. If it’s the latter, then the argument for excluding those subjects but including subjects with diabetes and hypertension needs to be re-examined.

*We agree with the concerns raised by the Reviewer. We performed a subgroup analysis in participants without hypertension or diabetes (Table 3) and we added the following statement in the revised version of our manuscript (Line 149-152).*

“Participants with epiretinal membrane and AMD with modification of the vascular architecture were excluded because they present primary modifications of the retinal vascular profile without systemic involvement.”
